# Excessive Vitamin E Supplementation: Implications for Ovarian Physiology and Reproductive Health

**DOI:** 10.1155/omcl/1330508

**Published:** 2026-01-22

**Authors:** Farzaneh Rabiee, Masoud Fattahi, Mohammad Iranzad, Mohsen Rahimi Andani, Farnoosh Jafarpour, Mohammad Hossein Sanei, Joel R. Drevet, Mohammad Hossein Nasr-Esfahani

**Affiliations:** ^1^ Department of Animal Biotechnology, Reproductive Biomedicine Research Center, Royan Institute for Biotechnology, ACECR, Isfahan, Iran, acecr.ac.ir; ^2^ Department of Pathology, Isfahan University of Medical Sciences, Isfahan, Isfahan Province, Iran, mui.ac.ir; ^3^ GReD Institute, EVALSEM, Faculty of Medicine, CRBC, University of Clermont Auvergne, Clermont-Ferrand, France, uca.fr

**Keywords:** antioxidants, fertility outcomes, infertility, redox stress, ROS, vitamin E

## Abstract

Infertility, a major global health problem affecting ~17.5% of couples, is influenced by various intrinsic (e.g., individual genetics) and extrinsic (e.g., related to environmental stimuli) factors. Oxidative stress and reactive oxygen species (ROS) are at the crossroads of these different stimulus–response pathways for both male and female gametes. While ROS are essential for ovarian processes such as folliculogenesis and oocyte maturation, changes in the ovarian ROS generation/recycling equilibrium can lead to impaired reproductive outcomes. Against this backdrop, noninvasive therapeutic approaches aimed at supplementing antioxidant (AO) molecules have emerged to correct prooxidant imbalances encountered in various stress situations. Numerous molecules have been tested, alone or in combination, for their beneficial effects on reproductive success in both men and women. The aim of this study was to investigate the effects of vitamin E supplementation at different levels on female reproductive performance and the molecular pathways involved. Groups of mice were treated with three different doses of vitamin E (optimal, overdose and severe overdose) and compared with control groups (no supplementation, sham groups [water and olive oil]). The results showed that both overdose and severe overdose of vitamin E showed significant reductions in pregnancy rates, litter size, and oocyte development capacity compared to the other groups. Blastocyst formation rates and quality were also significantly lower in these vitamin E overdosed groups, reflecting compromised embryonic quality. Severe vitamin E overdosage resulted in impaired folliculogenesis, with fewer antral follicles and corpora lutea and an increased number of atretic follicles. Notably, uterine thickness was significantly reduced in the severe vitamin E overdose group. Molecular analyses revealed increased GSH/GSSG ratios and higher ROS levels in granulosa cells. Intriguingly, in a context of increased ROS, we did not record any stimulation of the Nrf2 pathway and associated genes. A decrease in apoptosis in the ovarian environment marked by a lower Bax/Bcl2 ratio accompanied situations of vitamin E overdose. These findings shed new light on the consequences of excessive vitamin E intake and its implications for reproductive health. While optimal supplementation promotes fertility, excessive intake disrupts the redox balance, adversely affecting ovarian function and reproductive outcomes. This study highlights the importance of precise AO management to mitigate stress‐induced infertility and provides a framework for further research into the molecular mechanisms underlying vitamin E’s effects on ovarian physiology.

## 1. Introduction

Clinical infertility is defined as the inability to achieve pregnancy after 12 months or more of regular unprotected sexual intercourse [1]. According to the World Health Organization (WHO), infertility affects one in six couples worldwide [2]. Approximately one‐third of infertility cases are related to female factors, including hormonal disorders, endometriosis, pelvic inflammatory disease (PID), and polycystic ovarian syndrome (PCOS) [3–5]. Reactive oxygen species (ROS) play a critical role in the pathophysiology of female infertility, acting both as causative agents and as byproducts of ovarian dysfunction [6, 7]. Within the ovary, ROS function as secondary messengers that regulate essential signaling pathways involved in meiosis, ovulation, corpus luteum maintenance, and regression [8–11]. In the ovarian environment, a precise balance of ROS and antioxidants (AOs) supports normal physiological functions such as oocyte maturation, folliculogenesis, and hormonal regulation [8, 11–13]. Disturbances in this redox equilibrium have been implicated in impaired ovarian function and increased susceptibility to disorders such as PCOS [14, 15]. More specifically, during ovarian meiosis, precise regulation of the complex balance between ROS production and neutralization is essential for the arrest and resumption of oocyte development. Each month, a single dominant oocyte undergoes meiosis I, which is associated with high levels of ROS and attenuated AO production. Conversely, during meiosis II, primary enzymatic AOs such as catalase (CAT) and superoxide dismutase (SOD) are essential to protect the oocyte from excess ROS [11].

Counteracting ROS’s deleterious effects by stimulating AO capacity has logically emerged as a relevant potential therapeutic strategies [16]. When it comes to powerful AOs, vitamin E is an obvious choice. As a potent lipid‐soluble AO, it protects the plasma membrane from lipid peroxidation and disrupts the associated vicious cycle of ROS production.

Without surprise, vitamin E (often associated with vitamin C for its recycling) supplementation proved to be effective in protecting ovarian function and, more generally, fertility outcomes in females as in males [17–19]. In females, vitamin E supplementation has been reported to regulate cervical mucus production, enhance sperm viability, increase endometrial thickness, and contribute positively to gestation and fetal development [20–22]. Additionally, vitamin E improves insulin sensitivity and glucose tolerance, potentially benefiting women with metabolic disorders such as diabetes and PCOS [23, 24]. Despite these well‐documented benefits, the indiscriminate or excessive use of vitamin E supplementation raises concerns about disrupting the delicate redox balance, potentially leading to reductive stress with harmful effects comparable to oxidative stress [25, 26]. However, the specific consequences of vitamin E over dosage on ovarian physiology and reproductive performance remain inadequately explored. Therefore, the present study aims to investigate the effects of excessive vitamin E supplementation on ovarian function and fertility outcomes using a mouse model. We hypothesize that while physiological doses of vitamin E exert protective effects, its over dosage may disturb redox homeostasis and impair reproductive performance. This study seeks to fill a critical knowledge gap by defining the thresholds at which vitamin E shifts from being beneficial to detrimental. Thus providing essential insights for clinical recommendations on AO therapy in infertility.

## 2. Materials and Methods

### 2.1. Experimental Design

In this study, a total of 150 adult female NMRI mice (4 weeks old, 20–25 g) were obtained from the Royan Institute (Isfahan, Iran). They were randomly divided into six experimental groups (*n* = 25 per group). Groups included (1) control group (no supplementation and no manipulation), (2) a sham I group (gavage with water), (3) a sham II group (gavage with olive oil, as the vehicle for vitamin E administration), (4) an optimal vitamin E receiving 1000 mg/kg (OP‐VE), (5) overdose vitamin E receiving 2000 mg/kg (OD‐VE), and (6) a severe overdose of vitamin E receiving 4000 mg/kg (SOD‐VE).

**Figure 1:**
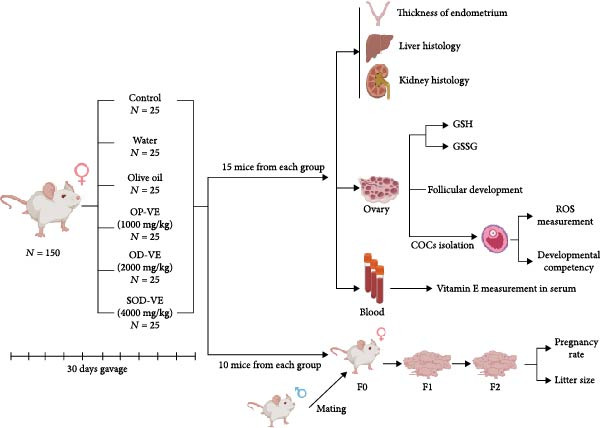
Experimental design and grouping of animals. A total of 150 adult female NMRI mice (20–25 g, 4 weeks) were randomly divided into six groups (*n* = 25 per group): (1) control (no supplementation and manipulation), (2) sham I (gavage with water), (3) sham II (gavage with olive oil), (4) optimal dose receiving 1000 mg/kg vitamin E (OP‐VE), (5) overdose receiving 2000 mg/kg vitamin E (OD‐VE) and (6) severe dose receiving 4000 mg/kg vitamin E (SOD‐VE). The mice were housed under controlled conditions with ad libitum access to food and water. After 30 days of supplementation, 15 mice per group were sacrificed for analysis, while the other 10 were mated to assess reproductive performance.

Mice were housed in the animal facility of the Royan Institute for Animal Biotechnology (Isfahan, Iran). They had free access to food and water and were maintained under controlled conditions (22 ± 2°C, with 45%–65% humidity and a 12‐h light/dark cycle). Vitamin E supplementation was carried out for a period of 30 days. All animal procedures were approved by the Institutional Animal Care and Use Committee (IACUC) and conducted in accordance with the American Veterinary Medical Association (AVMA) guidelines for euthanasia. Mice were anesthetized by intraperitoneal injection of ketamine (90 mg/kg body weight) and xylazine (10 mg/kg body weight). Adequate depth of anesthesia was confirmed by the absence of the paw withdrawal reflex. Once fully anesthetized, mice were euthanized by intracardiac exsanguination. Death was confirmed by cessation of breathing, cardiac arrest, and pallor of the mucous membranes. This protocol ensured that blood sampling and euthanasia were performed in a controlled manner under deep anesthesia. After euthanasia, 15 mice from each group were used for further analysis. The remaining 10 females from each group were mated with two males to assess their reproductive performance. (Figure 1: experimental design). The experimental design was approved by the Ethics Committee of the Royan Institute under the following registration number (IR.ACECR.AEC.1401.031).

### 2.2. Sample Collection and Assays

The body weight of each mouse was recorded at the start and at the end of the supplementation period, and weight gain was calculated as the difference between initial and final body weights. Blood samples were collected by cardiac puncture. Samples were then centrifuged at 2500 rpm for 15 min at room temperature to separate the serum, which was then frozen at −20°C until analysis. Serum vitamin E levels were analyzed using a commercial kit (vitamin A and E kit specifically for HPLC system) in accordance with the manufacturer’s instructions (Arvin Teb Co., Tehran, Iran). One ovary from each mouse was stored at −20°C for different assays, while the second ovary was preserved in 4% formalin for histological analysis. In addition to the ovaries, liver, kidney, and uterus tissues were also preserved in 10% formalin for future studies.

### 2.3. Histological Analysis

Fixed tissues were dehydrated, embedded in paraffin, and sectioned to a thickness of 5 μm. Sections were stained with hematoxylin and eosin (H&E) for morphological examination. Ovarian follicles were classified as primordial, primary, secondary, antral or atretic, using a light microscope (CX31 OLYMPUS, Japan) at ×400 magnification. Uterine endometrial thickness was measured using digital image analysis software (ImageJ) on images taken at 100x magnification. Histopathological evaluation of liver and kidney tissue was performed by a pathologist blinded to the treatment groups to identify any structural or pathological changes.

### 2.4. Pregnancy Rates and Litter Size

Pregnancies were confirmed by the presence of vaginal plugs, and the number of litters was recorded after parturition. Pregnancy rates were calculated as the percentage of pregnant mice in each group.

### 2.5. Oocyte Germinal Vesicle (GV) Collection and Granulosa Cell Isolation

The ovaries of 5 mice from each group were isolated and transferred to the laboratory for oocyte recovery and granulosa cell isolation. To recover the oocytes, the ovaries were mechanically punctured with a needle (29 gauge), causing the follicle to rupture and the oocytes to be released. The average number of GV oocytes recovered per mouse was calculated, and these GV oocytes underwent in vitro maturation (IVM) for in vitro fertilization (IVF) and in vitro culture (IVC) of embryos. In addition, released granulosa cells were isolated from the tissue remaining in the oocyte collection dishes to assess ROS levels.

### 2.6. IVM of Cumulus Oocytes Complexes (COCs)

Immature COCs were washed and transferred to droplets (20 µL) of maturation medium comprising *α*‐MEM supplemented with 5% FBS, hCG (7.5 IU/mL, Karma Pharmatech GmbH, Marburg, Germany), FSH (0.1 IU/mL, Cinnal‐F, CinnaGen, Tehran, Iran), streptomycin (50 µg/mL, Gibco, Invitrogen, Tehran, Iran), and penicillin G (75 IU/mL, Gibco, Invitrogen, Tehran, Iran), and coated with paraffin oil. After 18 h of incubation at 37.5°C in a humidified environment with ambient oxygen and 5% CO_2_, oocytes were matured to MII stage and were used for the IVF procedure.

### 2.7. Sperm Preparation and IVF

After ~18 h of IVM, the epididymis of a sacrificed male mouse was excised and placed in sperm‐active medium (GC302, Geneocell Ideal, Iran) for 45 min to recover cauda‐stored spermatozoa after repeated punctures with a needle. Then, about 10 µL of this solution was added to the previous drops of fertilization medium (G‐IVFTM PLUS Vitrolife, Gothenburg, Sweden) into which we had transferred the in vitro matured oocytes. After about 5 h, the zygotes were washed (HTCM 10% FBS) and cultured in GTL medium (G‐TLTM Vitrolife, Gothenburg, Sweden) for 4 days under 5% O_2_ and 6% CO_2_ at 37°C until they reached the blastocyst stage.

### 2.8. Differential Staining

For identification of inner cell mass (ICM) and trophectoderm (TE) cells, differential staining was performed. Day 4 blastocysts were collected and washed three times in PBS + PVA and permeabilized using 0.5% Triton‐X‐100 in HTCM containing 5 mg/mL bovine serum albumin for 30 s. Next, blastocysts were stained with 30 μg/mL propidium iodide for 10 s. Subsequently, blastocysts were incubated with 10 mg/mL Hoechst at 4°C for 15 min. Finally, blastocysts were mounted in light diagnostic mounting fluid (Cat. No. 5013; Merck Millipore [Burlington, MA]) and observed under a fluorescence microscope. ICM and TE were recognized on the basis of their blue and pink colors, respectively. Finally, the total number of cells (TCN: ICM + TE) was also calculated.

### 2.9. Assessment of ROS Levels and GSH/GSSG Ratio

To assess ROS levels in granulosa cells, the DCFHDA probe (2′, 7′‐ dichlorofuorescein, Sigma D6883) was used. Granulosa cells were centrifuged and washed twice with PBS. Cells were then trypsinized and filtered through 40 µm mesh to remove any residual ovarian connective tissue. Each sample comprised two centrifuge tubes (one as an unstained control and one test tube). Cells were stained with 10 µM DCFHDA for 30 min at 37°C in a humidified atmosphere of 5% CO_2_ in air, then washed again with PBS. Finally, the cells were resuspended in PBS and ROS levels detected using flow cytometry (BD CellQuestTM Pro) and expressed as the percentage of ROS‐positive cells and fluorescence intensity (in arbitrary units, AU) within granulosa cells. To assess redox status, the ratio of reduced glutathione (GSH) to oxidized glutathione (GSSG) in ovarian tissue was measured using commercial GSH/GSSG assay kits (NarGul‐Glutathione Assay Kit‐GSH and Naglut‐Glutathione Assay Kit‐GSSG) in accordance with the manufacturer’s instructions (Navand Salamat Co., Urmia, Iran).

### 2.10. Quantitative Real‐Time PCR (qRT‐PCR)

Total RNA was extracted from ovarian tissue by homogenizing 50 mg of ovarian tissue in 1 mL TRIzol reagent, following the manufacturer’s instructions (Yekta Tajhiz Azma, Iran). One µg of total RNA was treated with DNase I (Thermo Fisher Scientific, Waltham, MA, USA) to remove contaminating genomic DNA. cDNA was synthesized from total RNA using the Biotech rabbit cDNA Synthesis Kit, according to the manufacturer’s instructions (Biotechrabbit GmbH, Berlin, Germany). qRT‐PCR was performed with the fluorescent dye SYBR green (Yekta Tajhiz Azma, Iran) using the ABI system (Applied Biosystems StepOnePlus). All data were normalized against glyceraldehyde‐3‐phosphate dehydrogenase (GAPDH) and expressed using the 2^−*ΔΔ*Ct^ method. The primer list is provided in Table 1.

**Table 1:** List of primers used in this study.

Gene name	Sequences	*T*_m_
*Gapdh*	F 5′‐TGCCGCCTGGAGAAACC‐3′ R 5′‐TGAAGTCGCAGGAGACAACC‐3′	60

*Nrf2*	F 5′‐GCTCTCCATATTCCATTCC‐3′ R 5′‐TACCTCTCCTGCGTATATC‐3′	55

*Nqo1*	F 5′‐GCCAATCAGCGTTCGGTA‐3′ R 5′‐AGTTCATAGCATAGAGGTCAGA‐3′	62

*HO-1*	F 5′‐ATGTTGACTGACCACGAC‐3′ R 5′‐GCCCCACTTTGTTAGGAAA‐3′	56

*Bax2*	F 5′‐TTTTGCTACAGGGTTTCATC‐3′ R 5′‐GTCCAGTTCATCTCCAATTC‐3′	58

*Bcl2*	F 5′‐ACTTCTCTCGTCGCTACCGTC‐3′ R 5′‐AAGAGTTCCTCCACCACCGT‐3′	58

### 2.11. Western Blot

Tissues were lysed using TRI reagent (Yekta Tajhiz Azma, Iran), according to the manufacturer’s protocol. Equal amounts of solubilized proteins (30 μg) were subjected to electrophoresis on 12% SDS polyacrylamide gels. The separated protein fractions were transferred to a polyvinylidene difluoride (PVDF; Bio‐Rad, Hercules, CA) membrane. After blocking the membranes with 10% skimmed milk (Millipore, 115 363), the membranes were incubated with various primary antibodies for 2 h at room temperature. A monoclonal anti‐NRF2 (1:400, sc‐365949) and a monoclonal anti‐GAPDH (1:400, sc‐32233) were used. In the next step, membranes were washed (three times, 15 min) in PBS and incubated for 1 h at room temperature with horseradish peroxidase (HRP)‐conjugated anti‐mouse IgG (1:5000, sc‐516102) and anti‐rabbit IgG (1:5000, sc‐2357), respectively. HRP‐conjugated IgGs were visualized using an Amersham ECL Advance Western Blotting Detection Kit (GE Healthcare). The intensity of each band was quantified using ImageJ software (version 1.42Q; National Institutes of Health, Bethesda, MD, USA.

### 2.12. Statistical Analysis

Data are presented as mean ± SEM. Normality and homogeneity of variances were verified using the Shapiro–Wilk and Brown–Forsythe test, justifying the use of one‐way ANOVA. Multiple comparisons were corrected using the Benjamini–Hochberg (BH) method to control the false discovery rate, maintaining statistical power while limiting false‐positive findings. *p*‐values ≤0.05 were considered significant and are indicated as *p*  < 0.05,  ^∗∗^ *p*  < 0.01,  ^∗∗∗^ *p*  < 0.001, and  ^∗∗∗∗^ *p*  < 0.0001. Analyses were conducted using GraphPad Prism 9.0.0 (GraphPad Software, San Diego, CA, USA). As no significant differences were observed between control and sham groups, only comparisons between treatment and control groups are shown.

## 3. Results

### 3.1. Vitamin E Level in Serum

As shown in Figure 2A, only severe vitamin E overdose resulted in a significant increase in serum vitamin E levels compared to all other groups, with no significant differences observed among the other five groups.

Figure 2Effects of vitamin E supplementation on serum vitamin E levels and body weight gain in female mice. (A) Serum vitamin E levels in the different experimental groups, including control, sham groups (water and olive oil), optimal vitamin E dose (OP‐VE), overdose of vitamin E (OD‐VE) and severe overdose of vitamin E (SOD‐VE). Only the group that received a SOD‐VE showed a significant increase in serum vitamin E levels compared to all the other groups. No significant difference was observed among the other five groups. (B) Mean body weight gain in response to different doses of vitamin E supplementation. A significant reduction in mean body weight gain was observed only in the group that received OD‐VE, while no difference was recorded among the other groups. Data are presented as mean ± SEM. Comparison was made between groups. Significance is indicated as follows:  ^∗∗^ *p*  < 0.01,  ^∗∗∗^ *p*  < 0.001.

**Figure:**
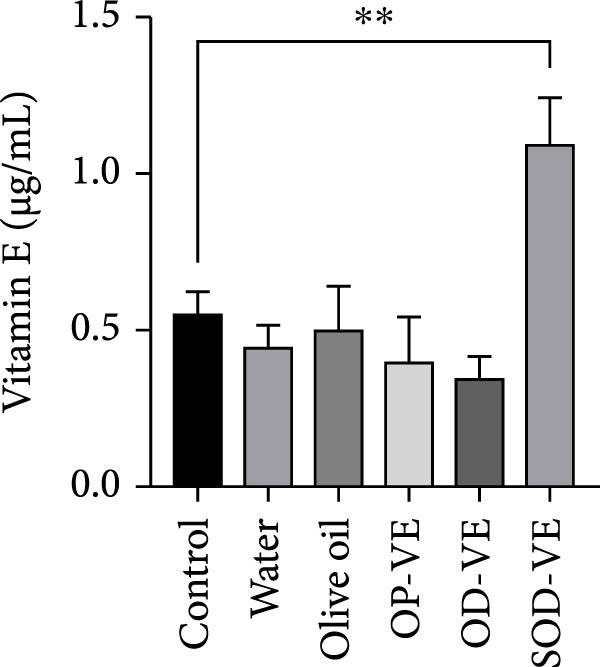
(A)

**Figure:**
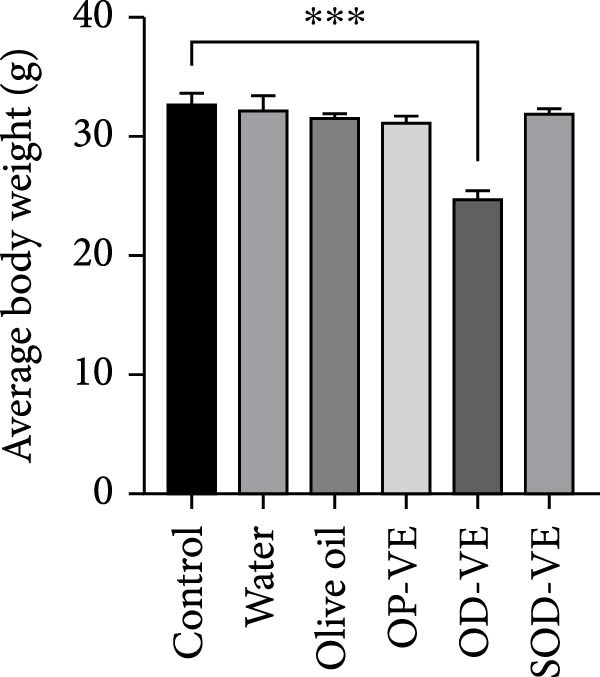
(B)

### 3.2. Vitamin E Supplementation and Weight Gain

In Figure 2B, a significant reduction in average body weight was observed only in the OD‐VE group compared with the control group and all other treatments (*p*  < 0.001). No statistically significant changes in body weight were detected among the control, water, olive oil, OP‐VE, and SOD‐VE groups.

### 3.3. Vitamin E Supplementation and Follicular Development

**Figure 3:**
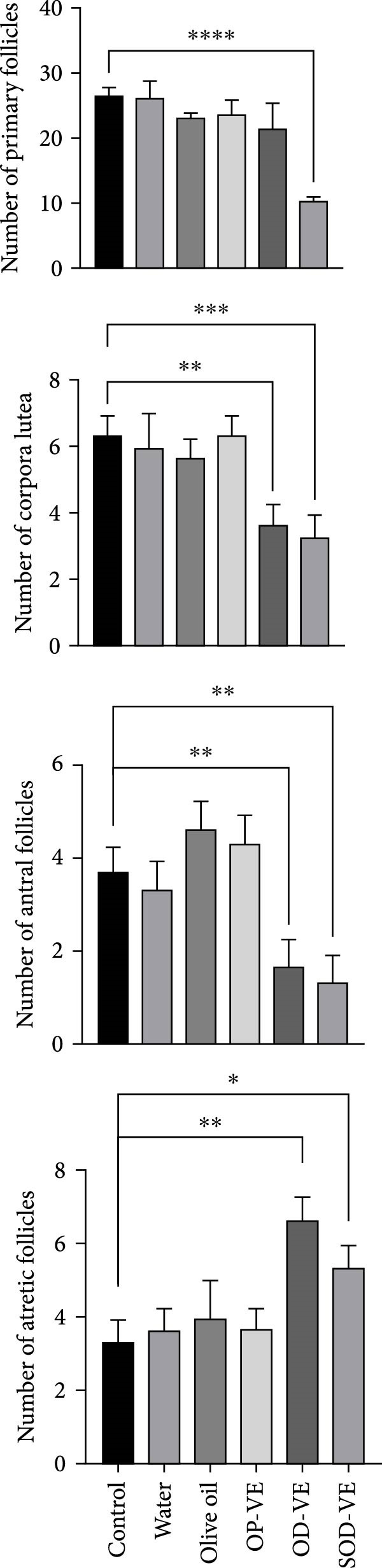
Effects of vitamin E supplementation on follicular development in female mice. The impact of varying doses of vitamin E on follicular development was evaluated by quantifying different types of follicles. The groups were defined as control, sham groups (water and olive oil), optimal vitamin E dose (OP‐VE), overdose of vitamin E (OD‐VE) and severe overdose of vitamin E (SOD‐VE). Only the group that received a severe overdose of vitamin E showed a significant reduction in the number of primary follicles compared to all the other groups. The number of antral follicles and corpora lutea was significantly reduced in the overdose and severe overdose groups, whereas no difference was observed among the other groups. In addition, the number of atretic follicles was significantly higher in the overdose and severe overdose groups than in the other four groups. Data are presented as mean ± SEM. A comparison was made between groups. Significance is indicated as follows:  ^∗^ *p*  < 0.05,  ^∗∗^ *p*  < 0.01,  ^∗∗∗^ *p*  < 0.001,  ^∗∗∗∗^ *p*  < 0.0001).

Figure 3 shows that only severe vitamin E overdose resulted in a significant reduction in the number of primary follicles. No significant differences were observed between the other five groups. With regard to the number of antral follicles and corpus luteum, only the two overdose groups (OD‐VE and SOD‐VE) showed a significant reduction in the number of these follicles compared with the other four groups, which showed no difference between each other. Furthermore, the number of atretic follicles increased significantly in these two groups compared with the other four groups.

### 3.4. Vitamin E Supplementation and Pregnancy Rates and Litter Size

As shown in Figure 4A, pregnancy rates were significantly reduced in the overdose and severe overdose groups compared with the other four groups. Similarly, the number of litters was also significantly lower in these two groups (Figure 4B). Gestation rate and litter size were also examined in subsequent generations, and no changes were observed (data not shown).

Figure 4Effects of vitamin E supplementation on pregnancy outcomes in female mice. The groups were defined as control, sham groups (water and olive oil), optimal vitamin E dose (OP‐VE), overdose of vitamin E (OD‐VE) and severe overdose of vitamin E (SOD‐VE). (A) Pregnancy rate in the different experimental groups. A significant reduction in pregnancy rates was observed in the overdose and severe overdose groups compared with the other four groups. (B) The average number of litters per pregnant mouse in each group. The groups that received an overdose and a severe overdose had a significantly lower number of litters than the other groups. Data are presented as mean ± SEM. Comparison was made between groups. Significance is indicated as follows:  ^∗^ *p*  < 0.05,  ^∗∗^ *p*  < 0.01,  ^∗∗∗^ *p*  < 0.001, and  ^∗∗∗∗^ *p*  < 0.0001.

**Figure:**
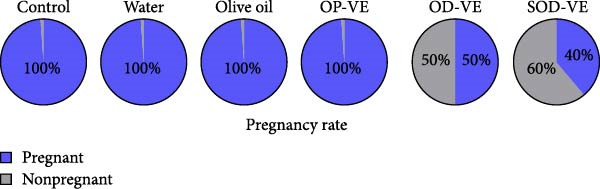
(A)

**Figure:**
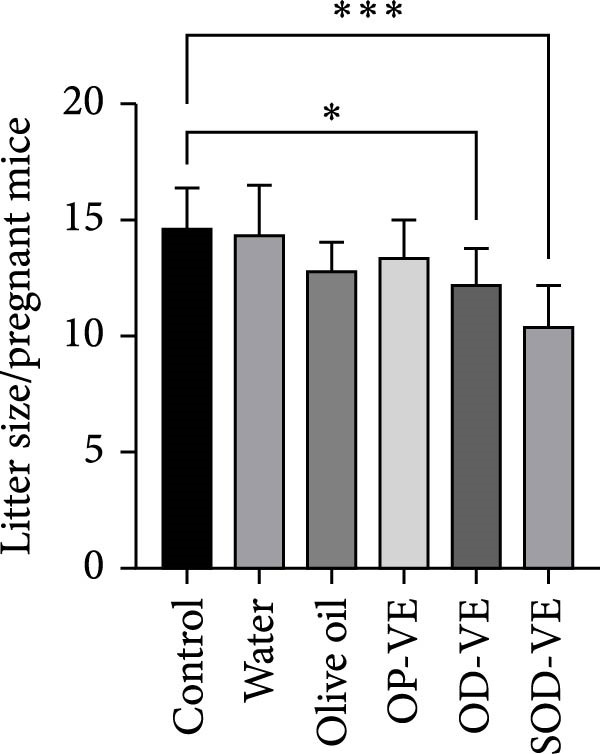
(B)

### 3.5. Vitamin E Supplementation and Oocyte Developmental Competency

The in vitro preimplantation development capacity of oocytes in all treatment groups was assessed. To this end, GV oocytes were harvested from female mice from each group and cultured for 18 h in IVM medium. Interestingly, the mean number of GV oocytes harvested *per* female mouse was significantly lower in the overdose and severe overdose groups. Although the mean number of GV oocytes recovered *per* female mouse was also reduced in the group supplemented with an optimal dose of vitamin E, this reduction did not appear to be significant (Figure 5A).

Figure 5Effects of vitamin E supplementation on in vitro preimplantation development of oocytes. The groups were defined as control, sham groups (water and olive oil), optimal vitamin E dose (OP‐VE), overdose of vitamin E (OD‐VE) and severe overdose of vitamin E (SOD‐VE). (A) The average number of germinal vesicle (GV) oocytes collected per female mouse. A significant reduction was observed in the (OD‐VE and SOD‐VE) groups compared to the other groups. Although a reduction was also observed in the group that received (OP‐VE) supplementation, this difference was not statistically significant. (B) Blastocyst formation rate after in vitro maturation, fertilization, and culture. The blastocyst formation rate was significantly lower in the (OD‐VE and SOD‐VE) groups compared to the other groups. (C, D) Total cell numbers in blastocysts derived from different experimental groups. The blastocysts from the (OD‐VE and SOD‐VE) groups had a significantly lower total number of cells compared to the other groups. Data are presented as mean ± SEM. Comparison was made between groups. Significance is indicated as follows:  ^∗∗^ *p*  < 0.01,  ^∗∗∗^ *p*  < 0.001,  ^∗∗∗∗^ *p*  < 0.0001.

**Figure:**
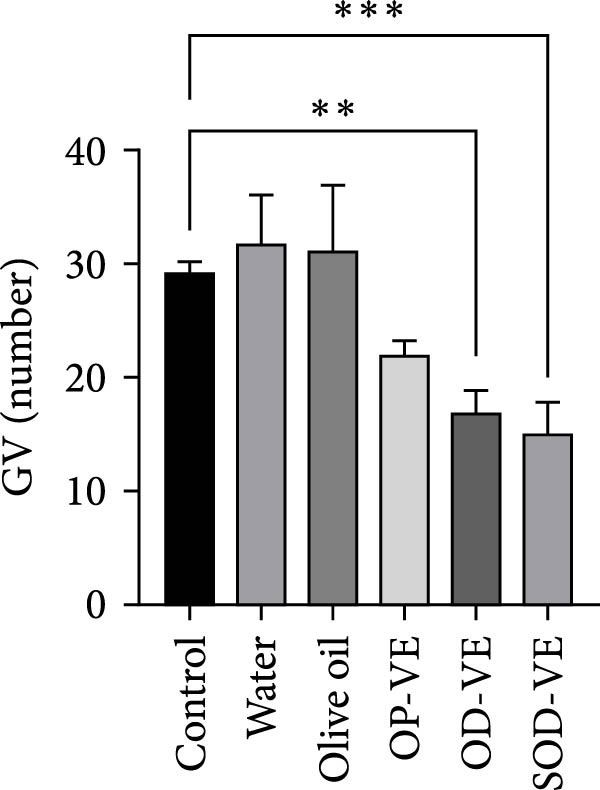
(A)

**Figure:**
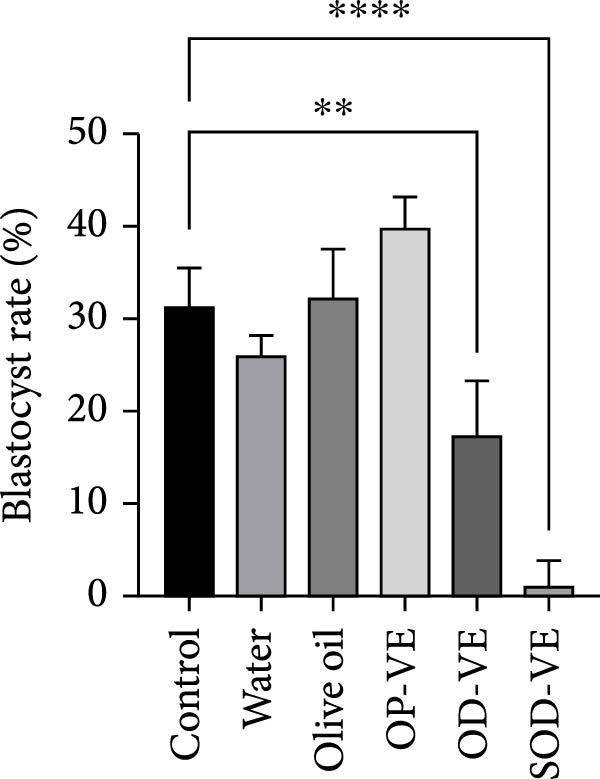
(B)

**Figure:**
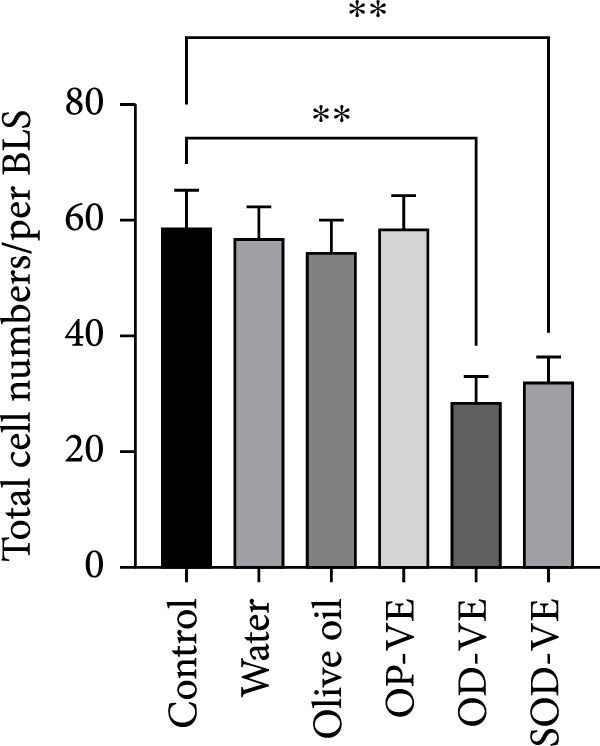
(C)

**Figure:**
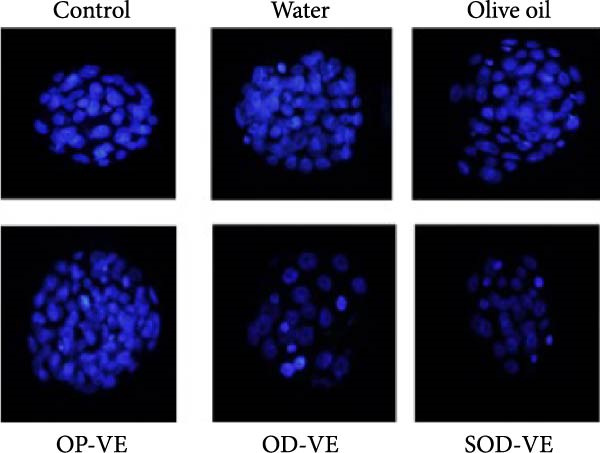
(D)

Mature oocytes were then subjected to IVF and IVC, and the rate of blastocyst formation was assessed. Figure 5B shows that the blastocyst rate in the overdose and severe overdose groups was significantly lower than the other groups (Figure 5B).

To further assess how vitamin E supplementation may affect the quality of derived blastocysts, differential staining was performed to determine the total cell number. Overall, the total cell number in blastocysts derived from the overdose and severe overdose groups was significantly lower than that in the other groups. (Figure 5C,D).

### 3.6. Vitamin E Supplementation and GSH/GSSG Ratio and ROS Level

Considering that an excess of AOs can eventually lead to reductive stress, we assessed the GSH/GSSG ratio as a marker of reductive stress in ovarian tissue in each group of mice. The results showed a significant increase in the GSH/GSSG ratio in the OD‐VE and SOD‐VE groups. The difference between this group and the sham groups was insignificant (Figure 6A). We also assessed the effect of vitamin E on granulosa ROS levels. The results revealed a significant increase in the percentage of ROS‐positive cells in the SOD‐VE group compared with the other five groups (Figure 6B).

Figure 6Effects of vitamin E supplementation on the reductive stress marker (GSH/GSSG) and ROS levels in ovarian tissue. The groups were defined as control, sham groups (water and olive oil), optimal vitamin E dose (OP‐VE), overdose of vitamin E (OD‐VE) and severe overdose of vitamin E (SOD‐VE). (A) Quantification of the GSH/GSSG ratio, as a reductive stress marker, in ovarian tissue from the different experimental groups. A significant increase in the GSH/GSSG ratio was observed in the OD‐VE and SOD‐VE groups, as well as in the OP‐VE group. (B) Flow cytometry analysis of ROS levels in granulosa cells. The percentage of ROS‐positive cells was significantly higher in the SOD‐VE group than in all other groups. Data are presented as mean ± SEM. Comparison was made between groups. Significance is indicated as follows:  ^∗^ *p*  < 0.05,  ^∗∗∗^ *p*  < 0.001,  ^∗∗∗∗^ *p*  < 0.0001.

**Figure:**
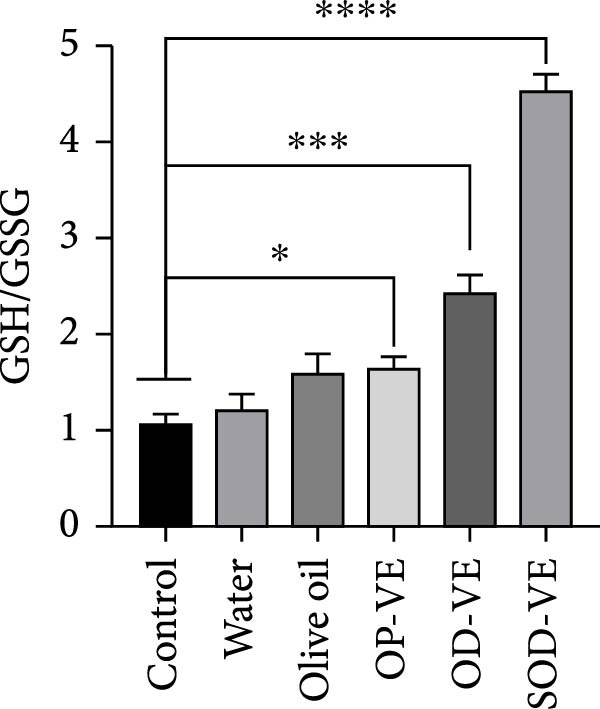
(A)

**Figure:**
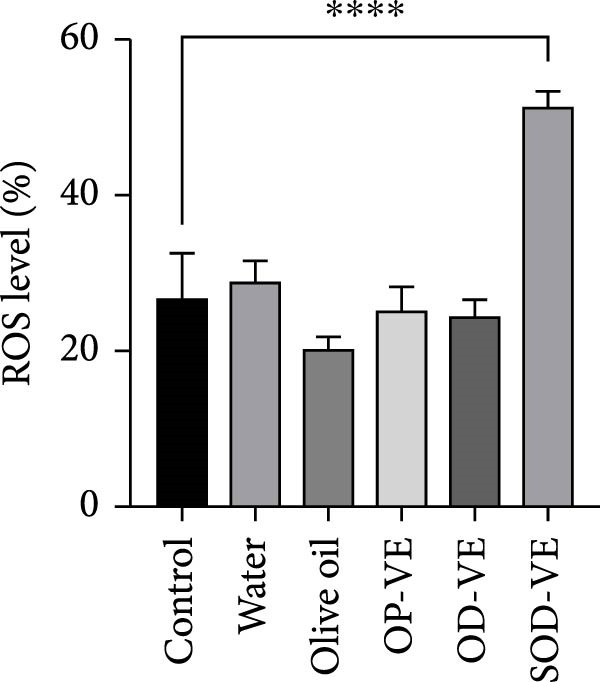
(B)

### 3.7. Vitamin E Supplementation and Uterine Thickness, Liver and Kidney Histology

As endometrial health and functionality are integral to fertility, we measured uterine endometrial thickness at the end of the treatment period (Table 2). Endometrial thickness was found to be significantly reduced in the SOD‐VE group compared with the control groups. Histological analysis of liver and kidney tissue from supplemented mice at all doses revealed no observable changes compared to control groups (data not shown).

**Table 2:** Endometrium thickness in mice treated with different concentration of vitamin E.

Experimental groups	Mean endometrium thickness (µm)	Adjusted *p*‐value
Water vs. control	333 ± 12	0.4439 ns
Olive oil vs. control	283 ± 15	0.1232 ns
OP‐VE vs. control	383 ± 34	0.0103 ^∗^
OD‐VE vs. control	266 ± 24	0.0251 ^∗^
SOD‐VE vs. control	216 ± 10	0.0007 ^∗∗∗^

*Note:* Endometrial thickness in the different experimental groups including control, sham groups (water and olive oil), optimal vitamin E dose (OP‐VE), overdose of vitamin E (OD‐VE) and sever overdose of vitamin E (SOD‐VE). Data are presented as mean ± SEM.

Abbreviation: ns, not significant.

*p*  < 0.05 ( ^∗^).

*p*  < 0.001 ( ^∗∗∗^).

### 3.8. Vitamin E Supplementation and Nrf2 Molecular Pathway

Although the Nrf2‐Keap1 pathway is recognized for its critical role in alleviating redox stress in mammals, our results showed no significant changes in Nrf2 gene expression, either at the mRNA or protein level (Figure 7A,B). Similarly, no significant changes were observed in the expression of downstream Nrf2 effectors, notably Nqo1 (NADPH dehydrogenase quinone 1) and HO‐1 (heme oxygenase 1, Figure 7C,D).

Figure 7Impact of vitamin E supplementation on the Nrf2‐Keap1 antioxidant pathway. The groups were defined as control, sham groups (water and olive oil), optimal vitamin E dose (OP‐VE), overdose of vitamin E (OD‐VE) and severe overdose of vitamin E (SOD‐VE). (A) Quantitative analysis of Nrf2 mRNA levels. (B) Corresponding levels of Nrf2 protein assessed by Western blot. (C) Expression of the downstream Nrf2 effector Nqo1 (D) and HO‐1. No significant differences were observed in Nrf2 expression (both mRNA and protein) or in Nqo1 and HO‐1 levels between the groups. Data are presented as mean ± SEM. Comparison was made between groups. ns, not significant.

**Figure:**
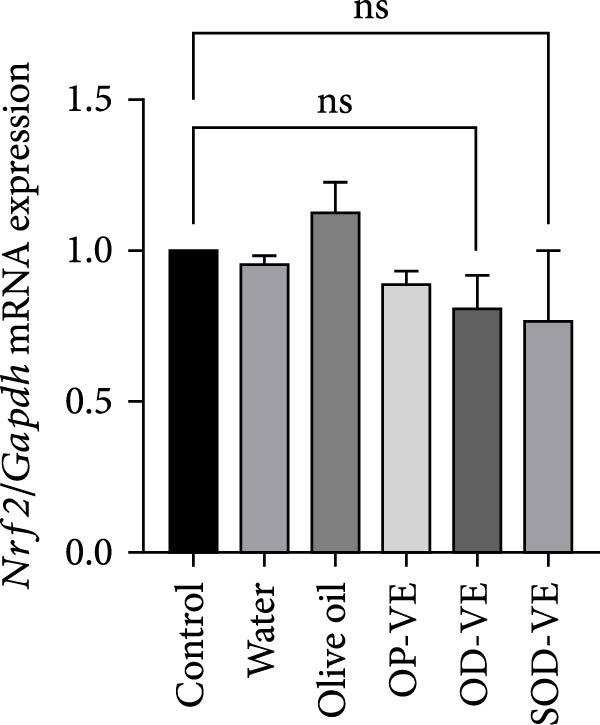
(A)

**Figure:**
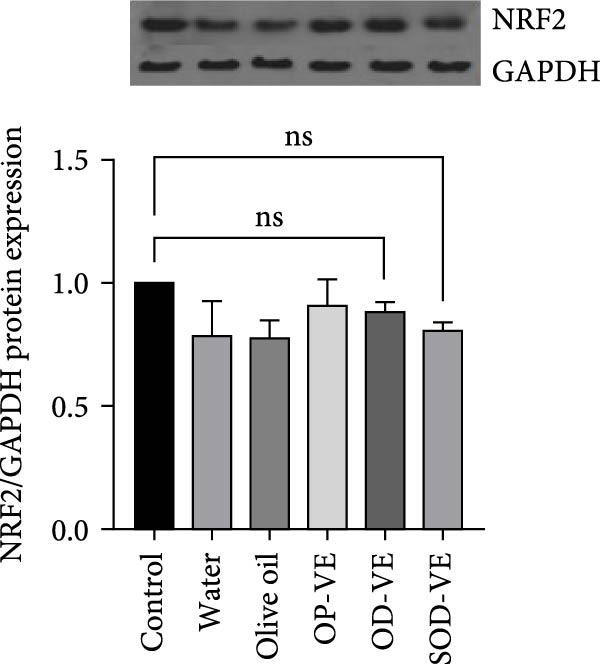
(B)

**Figure:**
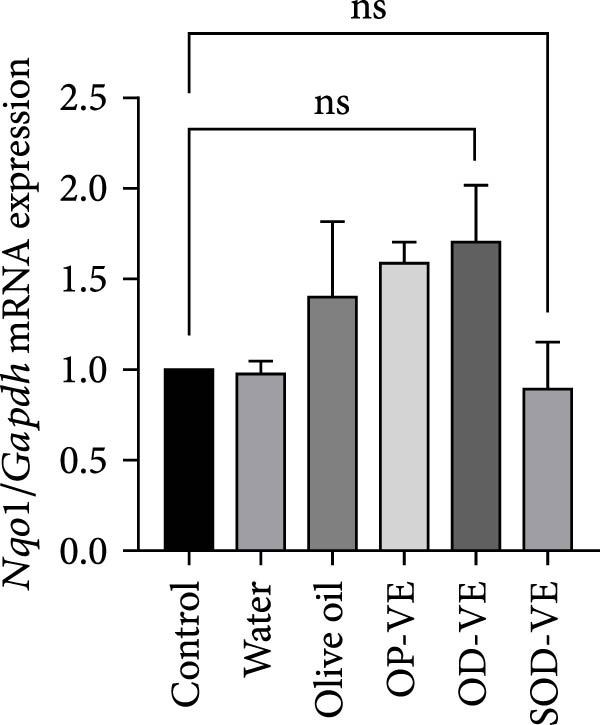
(C)

**Figure:**
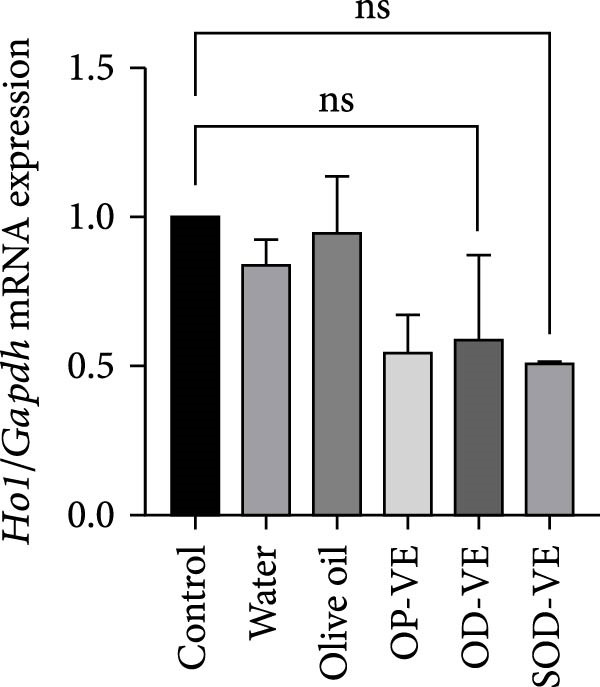
(D)

### 3.9. Vitamin E Supplementation and Apoptosis

In light of the observed increase in ROS levels, associated with the elevated GSH/GSSG ratio and the subsequent decline in folliculogenesis, activation of cell apoptosis could be a potential underlying pathway involved in the cellular response to excess of vitamin E. Our results showed a significant decrease in the proapoptotic Bax gene expression (Figure 8A) as well as the Bax/Bcl2 ratio (Figure 8C) with no significant change in the expression of the antiapoptotic Bcl2 effector (Figure 8B).

Figure 8Effects of vitamin E overdose on ovarian apoptotic markers. The groups were defined as control, sham groups (water and olive oil), optimal vitamin E dose (OP‐VE), overdose of vitamin E (OD‐VE) and severe overdose of vitamin E (SOD‐VE). (A) Relative expression of the proapoptotic Bax gene across experimental groups. A significant decrease in Bax gene expression was observed in the two overdose and sever overdose groups. (B) Relative expression of the antiapoptotic Bcl2 gene across experimental groups. No significant difference was detected, although a downward trend was observed in the overdose and severe overdose groups. (C) The Bax/Bcl2 ratio, an indicator of apoptotic potential, was significantly reduced in the overdose and severe overdose groups compared to the control group. Data are presented as mean ± SEM. comparison test to evaluate each group vs. control. Significance is indicated as follows:  ^∗∗^ *p*  < 0.01,  ^∗∗∗^ *p*  < 0.001; ns, not significant.

**Figure:**
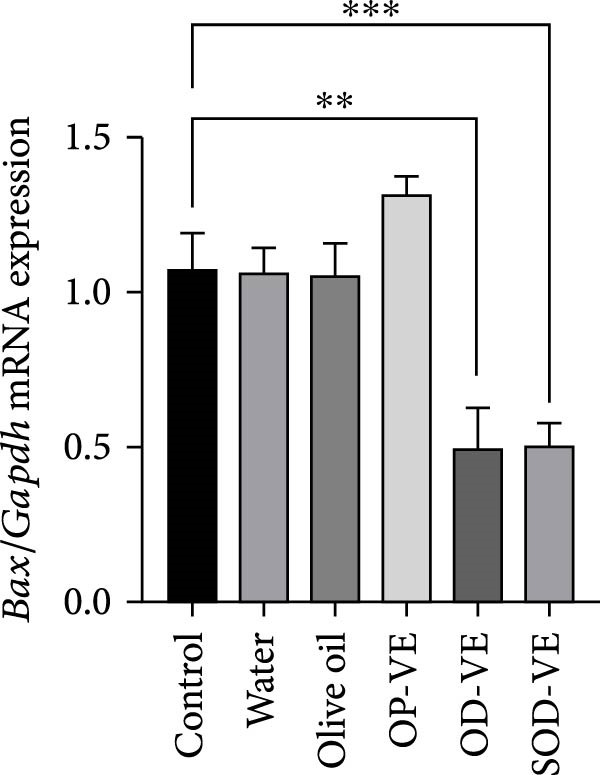
(A)

**Figure:**
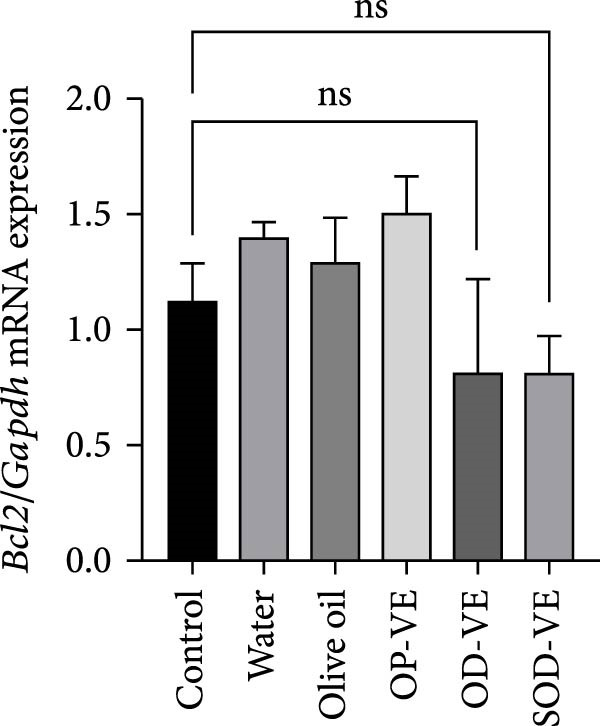
(B)

**Figure:**
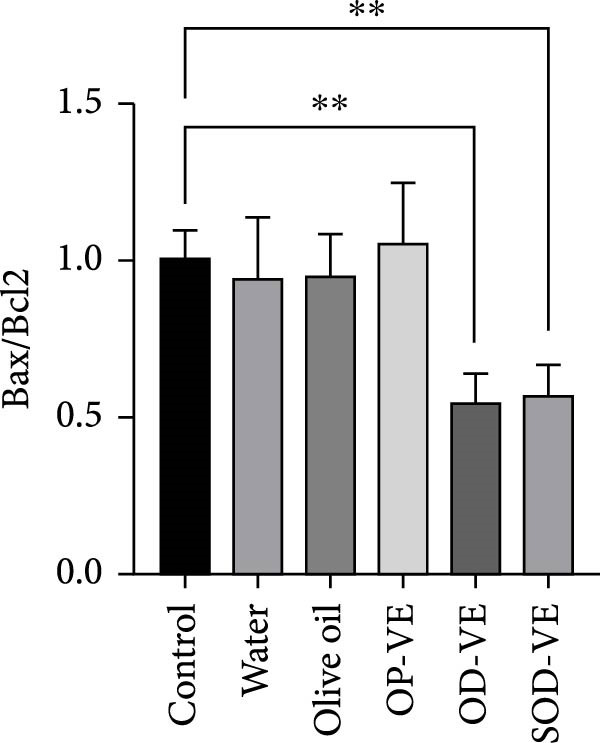
(C)

## 4. Discussion

In modern societies, particularly in the United States and Europe, over‐supplementation with vitamin E is a realistic concern given the widespread availability and aggressive marketing of dietary supplements. Vitamin E is often included in multivitamin formulations, fortified foods, and stand‐alone high‐dose capsules that can be purchased without medical supervision. Many individuals consume multiple overlapping products simultaneously, resulting in cumulative intakes that may exceed the recommended dietary allowance (RDA) by several fold. Public perception of AOs as inherently “beneficial” further contributes to indiscriminate use, especially among individuals seeking to improve fertility, slow aging, or enhance general well‐being. In addition, the absence of immediate toxic symptoms encourages chronic overuse, while the expanding practice of self‐medication in reproductive health amplifies this risk [27, 28].

Beyond reproductive concerns, chronic over‐supplementation may also predispose to long‐term adverse outcomes, including increased bleeding tendency, interactions with anticoagulant medications, disturbances in lipid metabolism, and possibly elevated risks of certain cancers, as suggested in some epidemiological studies. Prolonged exposure to excessive vitamin E may also perturb redox‐sensitive cellular signaling pathways, leading to subtle but cumulative effects on cardiovascular, neurological, and metabolic health. These potential long‐term risks underscore the need for carefully defined upper intake limits and for discouraging the routine use of high‐dose vitamin E supplements in the absence of clear clinical indications [29, 30]. Since the discovery of vitamin E in 1922, extensive research has highlighted its wide‐ranging biological functions, including protective effects against chronic diseases and roles in skin health, cardiovascular support, immunity, and aging. Understanding the molecular mechanisms and therapeutic applications of vitamin E remains an important focus in nutritional science. Nevertheless, its precise roles in reproductive physiology and female infertility are still under active investigation [31–33].

In the context of female infertility, vitamin E has been evaluated in both clinical and experimental settings, with evidence suggesting that it may enhance reproductive outcomes by reducing oxidative stress in the ovary, thereby improving ovulation and fertility in some patients. Vitamin E has also been reported to protect oocytes from oxidative damage and to improve endometrial receptivity, increasing the likelihood of successful implantation and pregnancy in women undergoing fertility treatments [34–36]. While most studies emphasize the benefits of vitamin E supplementation, far fewer have addressed the consequences of excessive intake. The indiscriminate use of “beneficial nutrients” often neglects Paracelsus’ principle that “every molecule is poison; it is only a question of dose.” Recent reports have raised concerns that high‐dose vitamin E may paradoxically impair reproductive function through the so‐called “antioxidant paradox,” wherein excess AOs disrupt cellular redox homeostasis and induce a pathological state known as reductive stress—an imbalance as detrimental as oxidative stress [37]. Excessive vitamin E intake has further been associated with increased bleeding risk, cardiovascular complications, and adverse drug interactions [38].

Against this background, the present study was designed to investigate the effects of vitamin E overdosing on ovarian physiology and female reproductive performance. To this end, female mice were supplemented for 1 month with vitamin E at doses of 1000, 2000, or 4000 mg/kg, representing optimal, overdose, and severe overdose regimens, respectively, based on previous reports [39–42]. Control and sham groups (water and olive oil) confirmed the absence of vehicle‐ or gavage‐related effects.

The results showed that optimal vitamin E supplementation conferred no significant effect over controls with respect to body weight, follicular growth, pregnancy rate, litter size, oocyte maturation, or blastocyst development. The only positive finding was a significant increase in endometrial thickness, consistent with clinical studies reporting improved endometrial receptivity following moderate supplementation [43, 44]. In contrast, both overdose and severe overdose regimens reduced endometrial thickness, indicating a dose‐dependent biphasic effect. Importantly, fertility rates declined sharply with excessive intake: pregnancy success was reduced to 50% in the overdose group and to 40% in the severe overdose group, accompanied by a significant reduction in litter size. Histological analyses confirmed impaired folliculogenesis, characterized by fewer antral follicles and corpora lutea, along with increased atresia, while in vitro assessments revealed marked reductions in oocyte maturation and blastocyst development.

These deleterious outcomes occurred despite largely stable serum *α*‐tocopherol concentrations, which remained within the physiological range (0.4–0.6 µg/mL) in both optimal and overdose groups, consistent with tight endogenous regulation and clearance mechanisms [39, 45]. Only severe overdosing produced a significant rise in serum vitamin E, suggesting that detoxification pathways had been overwhelmed. However, unchanged serum levels do not preclude tissue‐specific accumulation, and our findings support the hypothesis that reproductive tissues, particularly the ovary and endometrium, are highly sensitive to local vitamin E accumulation and redox imbalance [46, 47]. Redox analyses revealed that vitamin E supplementation significantly increased the ovarian GSH/GSSG ratio across all treatment groups, a marker of reductive stress. Contrary to expectations, this was not accompanied by upregulation of canonical Nrf2 target genes such as SOD, CAT, HO‐1, or Nqo1, in agreement with previous findings that vitamin E supplementation does not activate the Nrf2 pathway [41], These results suggest that alternative molecular mechanisms are responsible for the observed reductive stress.

One possibility is the induction of ferroptosis through iron dysregulation, as vitamin E overdose has been linked to ferroptotic processes in other tissues [48]. Another mechanism may involve disruption of the AO network: under normal conditions, vitamin C regenerates oxidized vitamin E in a process dependent on GSH. Excessive vitamin E may disturb this balance, leading to intracellular GSH accumulation, impaired redox cycling, and amplification of reductive stress [49]. Our observation of a reduced Bax/Bcl2 ratio in ovarian tissue further suggests an antiapoptotic shift, which may represent a compensatory pro‐survival response to redox imbalance.

An additional observation of interest was that vitamin E overdose, but not severe overdose, was associated with reduced body weight gain. This may reflect complex shifts between oxidative and reductive stress, both of which influence ROS dynamics, although further mechanistic studies are required to clarify this phenomenon. Collectively, our findings demonstrate that vitamin E overdose exerts profound negative effects on ovarian physiology and female reproductive performance, with detrimental consequences evident even in the absence of major changes in circulating vitamin E levels.

If extrapolated to humans, these results carry important clinical implications. Vitamin E is frequently recommended to reproductive‐age women, including infertile patients undergoing assisted reproductive technologies (ART). Our findings caution against routine or indiscriminate supplementation in the absence of confirmed deficiency, as excessive intake may compromise ovarian function, impair folliculogenesis, and reduce fertility. Future research should focus on quantifying tissue‐specific vitamin E accumulation, exploring the involvement of ferroptotic pathways, and investigating whether high‐dose supplementation alters hypothalamic–pituitary–ovarian axis regulation. Elucidating these mechanisms will be crucial to define safe upper limits of vitamin E intake and to refine clinical recommendations for its use in reproductive medicine.

In conclusion, excessive vitamin E intake induces reductive stress, disrupts ovarian physiology, and significantly compromises fertility in mice. These findings underscore the importance of dose precision and highlight the potential risks of indiscriminate supplementation, particularly in women seeking to optimize reproductive outcomes.

## Disclosure

All individuals who contributed to this work performed their duties as research academics or assistants at the Royan Institute. All facilities and services were provided by the Royan Institute. All scientific content and interpretations were prepared and verified by the authors.

## Conflicts of Interest

The authors declare no conflicts of interest.

## Author Contributions

**Farzaneh Rabiee**: conceptualization, project administration, supervision, writing – original draft, data curation. **Masoud Fattahi**: methodology, investigation, resources, visualization. **Mohammad Iranzad**: methodology, investigation, software. **Mohsen Rahimi Andani**: data curation, investigation. **Farnoosh Jafarpour**: validation, visualization, formal analysis. **Mohammad Hossein Sanei**: investigation, formal analysis. **Joel R. Drevet**: conceptualization, writing – review and editing. **Mohammad Hossein Nasr-Esfahani**: conceptualization, supervision, project administration, writing – review and editing. Farzaneh Rabiee and Masoud Fattahi contributed equally to this work.

## Funding

This study was privately funded by Masoud Fattahi and Mohammad Iranzad as part of their MSc project.

## Data Availability

The datasets generated during the current study are available from the corresponding author upon reasonable request.
